# Long-term outcomes of PARP inhibitors in ovarian cancer: survival, adverse events, and post-progression insights

**DOI:** 10.1016/j.esmoop.2024.103984

**Published:** 2024-11-13

**Authors:** V. Tuninetti, J.A. Marín-Jiménez, G. Valabrega, E. Ghisoni

**Affiliations:** 1 Department of Oncology, University of Turin, Medical Oncology, Ordine Mauriziano Hospital, Turin, Italy; 2 Department of Medical Oncology, Catalan Institute of Oncology (ICO), Barcelona, Spain; 3 Cancer Immunotherapy Group, Bellvitge Biomedical Research Institute (IDIBELL), Barcelona, Spain; 4 Department of Oncology, Immuno-Oncology Service, University Hospital of Lausanne (CHUV-UNIL), Lausanne, Switzerland; 5 Ludwig Institute for Cancer Research, Lausanne Branch, University of Lausanne (UNIL), Lausanne, Switzerland; 6 Agora Cancer Research Center, Lausanne, Switzerland

**Keywords:** ovarian cancer, PARP inhibitors, myelodysplastic syndrome, toxicity, overall survival, post-progression

## Abstract

Poly-ADP-ribose polymerase inhibitors (PARPis) have revolutionized the management of *BRCA*-mutated (*BRCA*^mut^) and homologous recombination deficiency (HRD)-positive ovarian cancer (OC). While long-term analyses clearly support the use of PARPi as maintenance therapy after first-line chemotherapy, recent data have raised concerns on detrimental overall survival (OS) in non-*BRCA*^mut^ OC, a greater incidence of myelodysplastic syndrome (MDS) and acute myeloid leukemia (AML), and unfavorable outcomes following subsequent platinum-based chemotherapy in pretreated OC patients. In this report we discuss the long-term follow-up results from phase III trials in pretreated OC patients, which led to the Food and Drug Administration’s withdrawal of PARPi indications in this setting. We summarize the newly available evidence concerning the risk of MDS/AML and the post-progression efficacy results after PARPi. We emphasize the importance of long-term follow-up and real-world data coming from international registries to define the efficacy and safety of stopping PARPi at relapse at a pre-specified time. To this point, biomarkers able to identify the patients who will experience long-term remission with PARPi maintenance or develop early resistance are urgently needed to guide treatment decision and duration.

## Highlights

• Long-term OS results support PARPi maintenance after first-line chemotherapy for both *BRCA*^mut^ or *BRCA*^wt^/HRD^pos^ patients.
• Detrimental OS results have been observed for PARPi use in later lines.
• MDS/AML incidence is higher when PARPis are used in later lines and it is associated with long-term exposure.
• Post-progression outcomes are needed to understand the clinical impact of PARPis in subsequent lines of treatment.

## Introduction

The use of poly-ADP-ribose polymerase inhibitors (PARPis) has transformed the care of advanced high-grade serous/endometrioid ovarian cancer (OC) patients. PARPis are currently indicated as maintenance treatment both in the first line and in the recurrent platinum-sensitive setting; therefore, most patients will receive PARPis at some point in their treatment journey.^1^^,^^2^

Recent data from PARPi use in the relapse setting raised some questions about efficacy, toxicities, and the optimal duration of treatment. In 2022, after matured detrimental data on overall survival (OS) derived from late-line studies of PARPi in OC, the Food and Drug Administration (FDA) withdrew the approval of niraparib, olaparib, and rucaparib as monotherapy in heavily pretreated OC, including platinum-resistant patients. The indications of niraparib and rucaparib as maintenance therapy at relapse were restricted to *BRCA*^mut^ patients. By contrast, the European Medicines Agency (EMA) did not withdraw the approval for *BRCA*^wt^ patients in this setting.^3^, ^4^, ^5^, ^6^

Importantly, while a similar toxicity profile for patients treated with PARPi in the first line and at relapse has been observed across all trials,^7^, ^8^, ^9^, ^10^ the attention of clinicians is now focused on the incidence of myelodysplastic syndrome (MDS) and acute myeloid leukemia (AML) which counted for ∼1% in the first-line setting and up to 8% at relapse.^11^ Due to the concern about higher risk rate of MDS and AML in latter lines, the detrimental survival, and the subsequent failure of platinum-based chemotherapy (PBC), the open question is how long we should continue PARPi at relapse.

## Long-term data on overall survival

Detrimental survival data for PARPi in OC were first observed for late lines of treatment in the SOLO-3 and ARIEL-4 trials.^12^^,^^13^ The SOLO-3 phase III study randomized platinum-sensitive germline *BRCA*^mut^ OC patients at relapse, after at least two prior lines of chemotherapy, to receive olaparib versus non-PBC treatment of physician’s choice. While the OS was similar between the olaparib and control groups in the intention-to-treat (ITT) population [hazard ratio (HR) 1.07, 95% confidence interval (CI) 0.76-1.49], a subgroup analysis of patients treated after three or more lines of therapy suggested a survival decrement for those receiving olaparib (HR 1.33, 95% CI 0.84-2.18).^12^ Similarly, the ARIEL-4 phase III trial randomized germline and/or somatic *BRCA*^mut^ OC patients after at least two previous lines of treatment to receive rucaparib monotherapy versus investigator’s choice. In the ITT population, OS favored the control arm with a median of 19.4 months in the rucaparib group versus 25.4 months in the chemotherapy group (HR 1.313, 95% CI 0.999-1.725). The difference in OS was driven by the subgroup of patients with platinum-resistant disease, with medians of 14.2 months and 22.2 months with rucaparib and chemotherapy, respectively (HR 1.511, 95% CI 1.053-2.170). In the patients with platinum-sensitive relapse, the median overall survival (OS) was 29.4 months with rucaparib versus 27.6 months with chemotherapy (HR 1.071, 95% CI 0.709-1.618).^14^ These finding led to the withdrawal by the FDA of single-agent olaparib and rucaparib indications for *BRCA*^mut^ heavily pretreated OC patients in August and June 2022, respectively.

More recently, the use of PARPi in the maintenance setting at platinum-sensitive recurrence showed detrimental OS data for niraparib and rucaparib in *BRCA*^wt^ patients.^13^^,^^15^^,^^16^ In particular, the NOVA and the ARIEL3 trials, which, respectively, randomized patients with platinum-sensitive relapse to receive niraparib or rucaparib versus placebo regardless of *BRCA* mutation, suggested a survival decrement in patients without germline *BRCA* mutation.^16^^,^^17^ The mOS in the NOVA trial was 31.1 months with niraparib and 36.5 months in the placebo arm (HR 1.10, 95% CI 0.831-1.459); in the ARIEL3 trial, rucaparib showed that survival was not improved in the experimental arm with a median of 36.0 months with rucaparib and 43.2 months with placebo (HR 0.995, 95% CI 0.809-1.223). Although no detrimental OS data were observed for BRCA^mut^ patients in both studies, no advantage was seen either (niraparib: HR 0.85, 95% CI 0.61-1.2; rucaparib: HR 0.86, 95% CI 0.99-1.7). Regarding olaparib, the SOLO2 phase III trial, which enrolled germline *BRCA*^mut^ patients with platinum-sensitive relapse to receive olaparib versus placebo, did not show any statistical advantage in terms of OS (HR 0.74, 95% CI 0.54-1.00) but olaparib provided an improvement of 12.9 months in mOS versus placebo.^15^

Therefore, due to the above-described results, the FDA approvals for both niraparib and rucaparib were restricted to *BRCA*^mut^ in the platinum-sensitive maintenance setting. By contrast, the EMA did not withdraw any indication acknowledging difficulties on interpreting OS data due to confounder factors such as post-progression cross-over, missing information on subsequent therapies, and insufficient statistical power for the OS analysis. Thus, both niraparib and rucaparib are still on label both in *BRCA*^mut^ and *BRCA*^wt^ patients in this setting).^18^ Finally, a recent meta-analysis did not find a statistically significant benefit of PARPi in this setting for unselected patients (HR 0.88, 95% CI 0.75-1.03). The explanation of these data can be also related to the high rate of cross-over (27% in NOVA, 46% in ARIEL3, and 38% in SOLO2, respectively).^19^ Moreover, it is important to highlight that these trials were not designed with OS as the primary endpoint. The primary endpoint for all three trials was progression-free survival (PFS), meaning the statistical analyses were underpowered to detect OS outcomes, and final conclusions should be interpreted with caution.

During the past few years, PARPi (olaparib, olaparib plus bevacizumab, niraparib and rucaparib) were approved also in the first line as maintenance therapy after PBC on the basis of the results of SOLO1, PAOLA-1, PRIMA, and ATHENA-mono phase III trials, respectively.^4^, ^5^, ^6^^,^^20^, ^21^, ^22^ The SOLO1 trial evaluated the efficacy of olaparib as maintenance therapy in patients with newly diagnosed advanced high-grade serous or endometrioid OC, *BRCA*^mut^, who had a complete or partial clinical response after PBC. The primary endpoint was PFS and the risk of disease progression or death was 70% lower with olaparib than with placebo (HR 0.30, 95% CI 0.23-0.41).^20^ At 7 years of follow-up, the HR for OS was 0.55 (95% CI 0.40-0.76); this means that 67.0% of olaparib patients versus 46.5% of placebo patients were alive, and 45.3% versus 20.6%, respectively, were alive and had not received a first subsequent treatment.^5^ The PAOLA-1 trial assessed the combination of olaparib plus bevacizumab maintenance versus bevacizumab alone in newly diagnosed OC. The primary endpoint was PFS. After a median follow-up of 22.9 months, the median PFS was 22.1 months for olaparib plus bevacizumab versus 16.6 months for bevacizumab (HR 0.59, 95% CI 0.49-0.72).^21^ The positive results were confirmed at a longer follow-up: mOS was 56.5 months versus 51.6 months in the ITT population (HR 0.92, 95% CI 0.76-1.12). In particular, in the BRCA^mut^ population mOS was 75.2 months and 66.9 months in the olaparib and placebo arms, respectively (HR 0.60, 95% CI 0.39-0.93); in the HRD-positive tumors (excluding those *BRCA*^mut^) mOS was not reached and 52.0 months (HR 0.71, 95% CI 0.45-1.13), and in the HRD-negative tumors mOS was 36.8 months and 40.4 months, respectively (HR 1.19, 95% CI 0.88-1.63).^4^ Finally, the PRIMA trial randomized newly diagnosed advanced OC to receive niraparib versus placebo as maintenance therapy after PBC. The primary endpoint was PFS. In the overall population, PFS was 13.8 months and 8.2 months (HR 0.62, 95% CI 0.50-0.76).^23^ Mature data on OS have been recently presented at the European Society of Medical Oncology and surprisingly showed no difference between niraparib and the control arm at 7 years of follow-up (HR 1.01, 95% CI 0.84-1.23). OS results were consistent in all pre-specified biomarker-defined subgroups (HRD and homologous recombination proficient population) and possibly affected by the threefold higher use of subsequent PARPi in the control arm than the niraparib arm.^24^

Finally, at the time of this publication, rucaparib is approved only by the EMA based on the positive results that emerged from the ATHENA-mono phase III study which also randomized newly diagnosed advanced OC patients to receive rucaparib versus placebo as maintenance therapy after PBC. Median PFS was 28.7 months with rucaparib versus 11.3 months with placebo in the HRD-positive population (HR 0.47, 95% CI 0.31-0.72); 20.2 months versus 9.2 months in the ITT population (HR 0.52, 95% CI 0.40-0.68); and 12.1 months versus 9.1 months in the HRD-negative population (HR 0.65, 95% CI 0.45-0.95).^22^ Data on OS are not mature yet.

In conclusion, long-term survival data further support the use of PARPi as maintenance strategy after PBC in the first line, where OS benefit has been demonstrated both in *BRCA*^mut^ and HRD-positive OC patients.

## Adverse side-effects of PARP inhibitors

Even if head-to-head comparisons of PARPi in randomized trials have not been carried out and only cross-comparison on the basis of the reported literature can be extrapolated, the three approved PARPis (niraparib, olaparib, rucaparib) share several common adverse effects (AEs) because of a class effect including nausea, fatigue, and anemia (Table 1).^25^ However, each PARPi also demonstrates some distinct AEs. Niraparib showed both in the PRIMA and NOVA trials a higher rate of hypertension and thrombocytopenia.^6^^,^^16^ Olaparib showed gastrointestinal toxicities in SOLO1 and SOLO2^26^^,^^27^ and rucaparib showed a higher rate of elevated transaminase in ATHENA-mono and ARIEL3.^9^^,^^22^ Finally, the safety profile of combination therapy with olaparib plus bevacizumab in PAOLA-1 was generally consistent with that observed with olaparib monotherapy, except for hypertension, which is commonly associated with bevacizumab.^4^ Of note, we should consider that patients included in clinical trials are often younger with a good performance status and less comorbidities than the real-world population, and hence, the potential benefits and AEs may not be superimposable.

**Table 1:** Data on toxicities observed in the phase III registrational trials of PARPi in ovarian cancer

PARPi	Trial	Setting	PARPi therapy duration	FU	G ≥ 3 TEAEs (%)	Most common G ≥ 3 TEAEs	Discontinuation rate due to TEAEs (%)	MDS/AML Patients, *n* (%)	
								PARP inhibitor	Placebo/TPC
Niraparib	PRIMA	First-line maintenance	3 years	24 months	70.5	Anemia 31% Thrombocytopenia 28.7% Neutropenia 12.8%	12	1/484 (0.2) BRCA status NA	0/244 (0)
	NOVA	Relapse maintenance	UPD, UT, PD	72 months	74.1	Thrombocytopenia 33.8% Anemia 25.3% Neutropenia 19.6%	14.7	9/136 (6.6) BRCA^mut^ 4/231 (1.7) BRCA^wt^	2/65 (3.1) BRCA^mut^ 1/114 (0.9) BRCA^wt^
Olaparib	SOLO1	First-line maintenance	2 years	88.9 months	39	Anemia 22% Neutropenia 9% Fatigue/asthenia 4%	12	4/260 (1.5) BRCA^mut^	1/130 (0.8) BRCA^mut^
	SOLO2	Relapse maintenance	UPD, UT, PD	65.7 months	36.9	Anemia 19.5% Neutropenia 5.1% Fatigue/asthenia 4.1%	10.8	16/195 (8.2) BRCA^mut^	4/99 (4) BRCA^mut^
	SOLO3	Relapse treatment	UPD, UT, PD	N/A	50	Anemia 21.3% Neutropenia 9.6% Thrombocytopenia 3.9%	7.3	NA	NA
Rucaparib	ATHENAmono	First-line maintenance	2 years	26.1 months	60.5	Anemia 28.7% Neutropenia 14.6% Increased AST/ALT 10%	11.8	2/425 (0.5) BRCA status NA	0/110 (0)
	ARIEL3	Relapse maintenance	UPD, UT, PD	51.4 months	60	Anemia 22% Increased AST/ALT 10% Neutropenia 8%	15	9/130 (6.9) BRCA^mut^ 5/245 (2.0) BRCA^wt^	3/63 (4.8) BRCA^mut^ 1/123 (0.8) BRCA^wt^
	ARIEL4	Relapse treatment	UPD, UT, PD	25 months	45	Anemia 20% Fatigue 8% Increased AST/ALT 8%	8	5/233 (2) BRCA status NA	0
Olaparib plus bevacizumab	PAOLA-1	First-line maintenance	2 years	22.9 months	57	Hypertension 19% Anemia 17% Neutropenia 6%	NA	6/535 (1) BRCA status NA	1/267 (0.4)

ALT, alanine transaminase; AML, acute myeloid leukemia; AST, aspartate transaminase; FU, follow-up; g, germline; G, grade according to CTCAE criteria; MDS, myelodysplastic syndrome; mut, mutated; *N*, number; NA, not available; PARPi, poly-ADP-ribose polymerase inhibitors; PD, patient decision; TEAEs, treatment-emergent adverse events; TPC, treatment of physician’s choice; UPD, until disease progression; UT, unacceptable toxicity; wt, wild-type.

With long-term outcome data available from trials both in upfront and recurrent settings, it is now clear that patients receiving PARPi are at increased risk of developing MDS and AML (Table 1) and these findings have now been expanded using pharmacovigilance data.^28^^,^^29^ Indeed, the risk of myeloid neoplasm in patients with OC is multifaceted and the frequent cytopenia occurring on PARPi therapy complicates the early identification of this serious hematological toxicity. Platinum-based therapy is associated with a significantly increased risk of clonal hematopoiesis with malignant driver mutations^30^ and there is a dose-dependent relationship between the risk of MDS and cumulative platinum exposure in patients with OC.^31^ *BRCA* mutation status is also relevant as germline *BRCA* variants are enriched in primary MDS/AML indicating that they may represent a germline predisposition (Table 1). Complex karyotypes and frequent TP53 mutations are commonly observed among these cases and the mortality of MDS and AML is higher compared to the general population.^29^ Data on MDS and AML in OC differ from trials carried out in the recurrent setting versus first-line trials (Table 1) with reported rates varying from 0.5 to 1.5% for PARPi use in the first line to up to 8% in the recurrence setting. Of note, the rate of MDS/AML was 11.4%, with *BRCA*^mut^ patients showing the highest percentage (15.2%) among patients who derived exceptional benefit from rucaparib maintenance.^11^^,^^32^^,^^33^ Two limitations should be considered when discussing these data: trials in the first line have an overall shorter follow-up than the trials at platinum-sensitive recurrence and, in addition, PARPi maintenance treatment after front-line chemotherapy is usually of a fixed duration (2 years for olaparib, olaparib plus bevacizumab and rucaparib, 3 years for niraparib). Real-world data confirm the actual risk of developing myeloid neoplasm in OC patients after chemotherapy and prolonged PARPi therapy.^34^ The management of these patients is complex, and outcomes are extremely poor. Therefore, biomarkers to identify patients at higher risk are urgently needed. The recent study carried out at the MD Anderson Cancer Center in 32 356 patients treated for any gynecological cancer from 2000 to 2022 confirmed a rate of secondary MDS/AML in the total population around 1%, consistent with prior reports, but a roughly 10-fold higher rate among OC patients treated with a PARPi (32 cases among 355 patients, 9%). A Multivariate analyses identified three significant factors: administration of neoadjuvant chemotherapy was associated with a protective effect, and low platelet counts during PARPi and a higher number of total carboplatin cycles were associated with secondary leukemia. Molecular analyses showed that 68% of the secondary leukemias in PARPi-treated patients had missense mutations in the DNA-binding domain of TP53.

Recent findings indicate that macrocytosis is a common feature in PARPi-associated anemia and red blood cell distribution width changes correlate with PARPi exposure.^22^ Modelling studies have identified red cell indices as predictive of a diagnosis of MDS.^23^ Delayed cytopenia after the first 3 months of starting PARPi with pancytopenia, bicytopenia, or thrombocytopenia may be an early safety signal and identify patients at potential risk of MDS.^24^ Clinicians should be alert to this possibility, treatment should be interrupted, and a hematologic consultation and bone marrow biopsy are advised in this case. Importantly, to date every patient starting PARPi therapy should be informed about the potential risk of developing MDS/AML, and the risks/benefits of stopping PARPi versus risk of MDS/AML especially for long-term exposure in the relapse setting need to be carefully assessed on a case-by-case basis.

Prospective studies are needed to systematically assess the effects of PARPi on pre-existing and chemotherapy-associated clonal hematopoiesis of indeterminate potential and the evolution toward MDS to stratify those patients at greatest risk and inform oncological treatment decisions.

## Response to subsequent therapy

**Figure 1:**
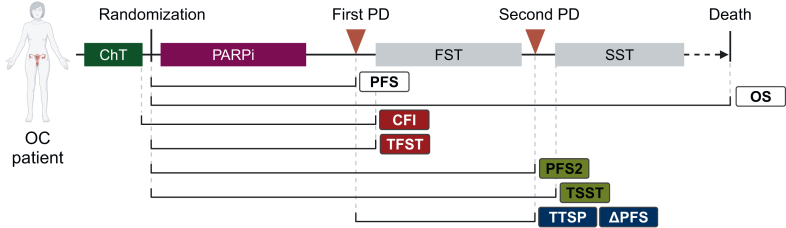
**Post-progression endpoints in PARPi clinical trials.** Diagram of different post-progression outcomes evaluating treatment effect on delaying subsequent treatment (CFI, TFST; in red), the added value of subsequent treatment (PFS2 and TSST; in green), or subsequent treatment efficacy from date of progression (TTSP and ΔPFS, in blue). ΔPFS, PFS2-PFS; CFI, chemotherapy-free interval; ChT, chemotherapy; FST, first subsequent therapy; OC, ovarian cancer; OS, overall survival; PARPi, poly-ADP-ribose polymerase inhibitors; PD, progressive disease; PFS, progression-free survival; PFS2, second progression-free survival; SST, second subsequent therapy; TFST, time to FST; TSST, time to SST; TTSP, time to second progression. Created with BioRender.

The incorporation of PARPi as first-line maintenance treatment leads to the fact that nearly all the *BRCA*^mut^ and HRD-positive, and a relevant proportion of HRD-negative, recurring or progressing OC patients will have been exposed to a PARPi. Of them, ∼85% will remain progression free after 6 months of PARPi initiation, thus being eligible for subsequent PBC in case of recurrence.^4^^,^^6^^,^^22^^,^^35^ Furthermore, the recently published phase III OReO clinical trial (discussed in the following text) has opened the possibility for these patients to be retreated with maintenance PARPi following chemotherapy.^36^ In view of that, understanding the impact of PARPi maintenance on subsequent therapies is of main relevance. With this objective, different post-progression outcomes have been reported in PARPi trials so far regarding first subsequent treatment (FST) delay and efficacy (Figure 1).

Despite the reported improvement in post-progression outcomes in clinical trials evaluating PARPi as maintenance for recurrent OC (Table 2, relapse maintenance) recent data raised a general concern on the potential effect of PARPi on subsequent PBC efficacy. Initial data on post-progression treatment efficacy came from a real-world study of recurrent *BRCA*^mut^ patients, where olaparib-treated patients showed a lower-than-expected response to chemotherapy, suggesting potential cross-resistance between PARPi and subsequent therapy.^37^ An exploratory analysis of the SOLO2 trial evaluated FST by treatment modality and suggested that the efficacy of chemotherapy is reduced after olaparib maintenance.^38^ Indeed, in this study, the patients treated with PBC after olaparib showed shorter time to second progression (TTSP) compared with the placebo group, although short follow-up and potential selection biases must be acknowledged.^39^ Conversely, the time between first and second progression (ΔPFS) was similar between PARPi and placebo in the ARIEL3 and NOVA trials, although differences between platinum-based and non-platinum-based subsequent regimens were not reported.^7^^,^^40^^,^^41^ More recently, other real-world retrospective studies have shown impaired subsequent chemotherapy efficacy in cohorts of patients progressing to PARPi.^42^, ^43^, ^44^, ^45^ Notably, both platinum-sensitive and -resistant patients were evaluated in these studies, and the PARPi and FST regimens varied, making it difficult to draw specific conclusions by patient subgroup or treatment protocol. *BRCA*^mut^ patients seemed to be more sensitive to the detrimental effects of PARPi^43^^,^^44^ while BRCA status was not associated with survival outcomes in a recent study^45^; so understanding the impact of *BRCA*^mut^/HR status on FST efficacy is still to be deciphered. Overall, those patients progressing to PARPi maintenance in the recurrent setting can respond to subsequent chemotherapy, but they are likely less responsive to PBC compared to patients not treated with PARPi. In our view, the concept of platinum sensitivity in this setting should be revisited and multi-centric and well-stratified studies are mandatory to understand and anticipate clinical outcomes of OC patients after post-PARPi progression.

**Table 2:** Post-progression outcomes reported in PARPi maintenance clinical trials

PARPi	Study	Population/control arm	Post-progression endpoints				
			CFI	TFST	PFS2	TSST	TTSP/ΔPFS
Relapse maintenance							
Niraparib	NOVA^7^ (presented at SGO 2023)	*N* = 553, all comers/placebo	gBRCA^mut^: 20.0 versus 9.4; HR 0.39 (0.27-0.56) gBRCA^wt^: 13.4 versus 8.7; HR 0.56 (0.43-0.73)	gBRCA^mut^: 19.1 versus 8.6; HR 0.57 (0.41-0.78) gBRCA^wt^: 12.4 versus 7.4; HR 0.58 (0.45-0.74)	gBRCA^mut^: 29.9 versus 22.7; HR 0.70 (0.50-0.97) gBRCA^wt^: 19.5 versus 16.1; HR 0.80 (0.63-1.02)	gBRCA^mut^: 29.7 versus 19.7; HR 0.63 (0.45-0.88) gBRCA^wt^: 20.3 versus 16.7; HR 0.84 (0.65-1.08)	NA^a^
Olaparib	Study19^49^^,^^50^	*N* = 265, all comers/placebo	NA	ITT: 13.3 versus 6.7; HR 0.39 (0.30-0.52) BRCA^mut^: 15.6 versus 6.2; HR 0.33 (0.22-0.49)	NA	ITT: 19.1 versus 14.8; HR 0.53 (0.40-0.69) BRCA^mut^: 21.4 versus 15.3; HR 0.43 (0.29-0.64)	NA
	SOLO2^27^^,^^38^^,^^51^	*N* = 295, BRCA^mut^/placebo	NA	ITT: 27.4 (22.6-31.1) versus 7.2 (6.3-8.5); HR 0.37 (0.28-0.48)	ITT: NR versus 18.4; HR 0.50 (0.34-0.72)	ITT: 35.8 (29.4-43.9) versus 18.9 (15.5-21.5); HR 0.51 (0.39-0.68)	Treated after PD: All: 6.9 versus 12.1; HR 2.17 (1.47-3.19) PBC: 7.0 versus 14.3; HR 2.89 (1.73-4.82) Non-PBC: 6.0 versus 8.3; HR 1.58 (0.86-2.90)
Rucaparib	ARIEL3^40^	*N* = 564, all comers/placebo	ITT: 14.3 (13-17.4) versus 8.8 (8-10.3); HR 0.43 (0.35-0.53) BRCA^mut^: 20.8 (17.7-27.8) versus 8.7 (7.2-10.9); HR 0.28 (0.19-0.41) HRD: 18 (14.3-19.4) versus 9.1 (8-10.8); HR 0.40 (0.31-0.53)	ITT: 12.4 (11.1-15.2) versus 7.2 (6.4-8.6); HR 0.43 (0.35-0.52) BRCA^mut^: 18.9 (15.9-25.3) versus 7.2 (5.5-9.1); HR 0.28 (0.20-0.41) HRD: 16.4 (12.5-17.9) versus 7.4 (6.5-9.1); HR 0.39 (0.30-0.51)	ITT: 21 (18.9-23.6) versus 16.5 (15.2-18.4); HR 0.66 (0.53-0.82) BRCA^mut^: 26.8 (23.4-41.4) versus 18.4 (15.7-23.6); HR 0.56 (0.38-0.83) HRD: 25.3 (21.9-28.5) versus 18.4 (15.8-22.1); HR 0.66 (0.49-0.87)	ITT: 22.4 (19.1-24.5) versus 17.3 (14.9-19.4); HR 0.68 (0.54-0.85) BRCA^mut^: 28.8 (24.4-34.2) versus 17.7 (15.1-21.6); HR 0.53 (0.36-0.80) HRD: 26.2 (22.9-30.6) versus 19 (15.8-21.7); HR 0.67 (0.50-0.91)	NA^a^
First-line maintenance							
Niraparib	PRIMA (presented at ESMO 2024)^24^	*N* = 733, all comers (high-risk)/placebo	NA	ITT: 17 versus 12; HR 0.74 (0.62-0.89) HRD: 26.9 versus 13.9; HR 0.55 (0.43-0.71) HRP: 11.6 versus 7.9; HR 0.88 (0.65-1.18)	ITT: 30.1 versus 27.6; HR 0.96 (0.79-1.17) HRD: 43.4 versus 39.3; HR 0.87 (0.66-1.17) HRP: 21.4 versus 18.1; HR 0.90 (0.66-1.23)	NA	NA
	PRIME^52^	*N* = 384, all comers (China)/placebo	NA	ITT: 29.2 (22.4-NR) versus 11.9 (8.8-14.8); HR 0.45 (0.34-0.59)	NA	NA	NA
Olaparib	SOLO1^5^	*N* = 39, BRCA^mut^/placebo	NA	ITT: 64 (47.7-93.4) versus 15.1 (12.7-20.5); HR 0.37 (0.28-0.48)	NA	ITT: 93.2 (84.2-NR) versus 40.7 (32.9-54.4); HR 0.50 (0.37-0.67)	NA
Rucaparib	ATHENA-MONO (presented at SGO 2024)	*N* = 538, all comers/placebo	NA	ITT: 23.3 versus 12.1; HR 0.52 (0.40-0.67) HRD: 32.7 versus 15.1; R 0.50 (0.33-0.76)	ITT: 36 versus 26.8; HR 0.84 (0.63-1.13) HRD: NR versus 39.9; HR 0.75 (0.46-1.24)	NA	NA
Olaparib and bevacizumab	PAOLA-1^46^^,^^47^	*N* = 806, all comers/placebo and bevacizumab	NA	NA	ITT: 36.5 versus 32.6; HR 0.78 (0.64-0.95) HRD: 50.3 versus 35.3; HR 0.56 (0.41-0.77) HRP: 24.4 versus 26.4; HR 1.04 (0.77-1.42)	ITT: 38.2 versus 31.5; HR 0.78 (0.64-0.95) HRD: NR versus 35.2; HR 0.48 (0.35-0.66) HRP: 27.1 versus 29.1; HR 1.05 (0.82-1.36)	NA^b^^,^^c^

Median duration in months and the hazard ratio (HR) (with 95% confidence intervals in brackets) for the comparison PARPi versus control arm are shown for all the endpoints.

ΔPFS, PFS2-PFS; CFI, chemotherapy-free interval; FST, first subsequent treatment; gBRCA^mut^, germline-BRCA mutated; HRD, homologous recombination deficient; HRP, homologous recombination proficient; ITT, intention-to-treat population; mut, mutated; NA, not available; NR, not reached; PARPi, poly-ADP-ribose polymerase inhibitors; PBC, platinum-based chemotherapy; PD, patient decision; PFS2, second progression-free survival; SST, second subsequent treatment; TFST, time to FST; TSST, time to second subsequent therapy; TTSP, time to second progression; wt, wild-type.

a Exploratory analysis showed not significantly different PFS2-PFS between treatment groups and across all the study cohorts.^7^^,^^41^

b Harter et al. reported time from FST to SST for those patients who progressed and were treated with chemotherapy.^47^

c Marth et al. reported time from FST to SST for those patients who progressed and were treated with PBC followed or not by PARP maintenance.^48^

In the first-line scenario, favorable yet immature post-progression outcomes have been reported for PARPi-treated patients compared to placebo (Table 2, first-line maintenance). A real-world data study showed that PARPi maintenance was associated with worse response to FST and independently predicted shorter TTSP after subsequent chemotherapy (presented at SEOM2024). Post-progression data are also available from the PAOLA-1 trial, confirming the sustained benefit of the olaparib–bevacizumab regimen for the *BRCA*^mut^/HRD population beyond progression.^46^ Two *post hoc* exploratory analysis evaluated FST efficacy in those patients progressing after PAOLA-1.^47^^,^^48^ In the first one, subsequent chemotherapy efficacy was similar between patients progressing after treatment in both groups (olaparib–bevacizumab versus placebo–bevacizumab). However, it appeared to be dependent on the time of progression for PARPi-treated patients, with impaired efficacy in those patients progressing during compared to after olaparib treatment.^47^ The second study evaluated the subset of patients treated with PBC, followed or not by PARPi rechallenge, as FST. Time to second subsequent therapy (TSST) was also superior in those patients progressing after PARPi completion. Consistent with the OReO results, patients treated with PBC followed by PARPi rechallenge had better outcomes compared to those treated with PBC alone.^48^ Considering this, distinguishing between patients who progress under or after PARPi will be particularly relevant in the first-line setting, where treatment duration is limited, as the expected response to subsequent therapies may differ substantially.

The OReO phase III clinical trial^36^ evaluated olaparib maintenance for platinum-sensitive recurrent OC patients who have received one prior PARPi and were in response following their last PBC regimen. OReO is the first study to demonstrate a significant yet modest PFS benefit of PARPi rechallenge versus placebo maintenance, regardless of the *BRCA* mutation status [median PFS 4.3 versus 2.8 months (HR 0.57, 95% CI 0.37-0.87) in *BRCA*^mut^ patients; and 5.3 versus 2.8 months (HR 0.43, 95% CI 0.26-0.71) in BRCA^wt^ patients]. Regarding the effect of PARPi on subsequent therapies, some important lessons can be drawn from this trial. Firstly, prior exposure to a PARPi did not appear to preclude some benefit from PARPi rechallenge, at least for this selected population, who was required to demonstrate sensitivity to prior PARPi (see OReO inclusion criteria) and to be platinum sensitive. Secondly, with comparable TSST in both cohorts (*BRCA*^mut^ and non-*BRCA*^mut^), PARPi rechallenge did not seem to impact the efficacy of the subsequent therapy. Finally, very few patients received prior PARPi as first-line maintenance, so the benefit in this scenario and its dependence on the timing of progression (during or after PARPi) remains uncertain.

The observed detrimental effect of PARPi on subsequent PBC efficacy in the relapse setting should be formally explored in the first-line maintenance scenario. While more mature post-progression data are expected, collective efforts are needed to include post-progression outcomes and pre-specified subgroup analysis in upcoming studies.

## Conclusion

With the incorporation of PARPi as the standard-of-care maintenance in the front line, the effect of PARPi in subsequent lines is becoming less relevant (at least in the countries where PARPis are available in the front line). However, the emerging data on detrimental OS at relapse, the higher rate of MDS/AML for patients undergoing PARPi therapy for a long time (especially for *BRCA*^mut^), and the poor response rate to PBC after progression to PARPi raise doubts on the optimal duration of PARPi treatment at relapse. One option would be to consider the use of PARPi for a certain duration, as currently approved for first-line maintenance; the question is, for how long? To answer this, real-world data and international registry led by academic institutions and multi-centric working groups are urgently needed. Importantly, post-progression outcomes need to be clarified and homogenized across trials. The optimal strategy for recurrent OC patients who relapse after PARPi remains one of the challenges for the near future. Randomized trials which include post-PARPi endpoints and translational objectives are needed to decipher mechanisms of resistance to PARPi and delineate the optimal treatment sequence for our patients.
